# Cardiovascular Mechanisms of Action of Anthocyanins May Be Associated with the Impact of Microbial Metabolites on Heme Oxygenase-1 in Vascular Smooth Muscle Cells

**DOI:** 10.3390/molecules23040898

**Published:** 2018-04-13

**Authors:** Emily F. Warner, Ildefonso Rodriguez-Ramiro, Maria A. O’Connell, Colin D. Kay

**Affiliations:** 1 Department of Nutrition and Preventative Medicine, Norwich Medical School, Bob Champion Research and Education Building, University of East Anglia, Norwich NR4 7UQ, UK; emily.warner@uea.ac.uk (E.F.W.); I.Rodriguez-Ramiro@uea.ac.uk (I.R.-R.); 2 School of Pharmacy, University of East Anglia, Norwich NR4 7TJ, UK; m.oconnell@uea.ac.uk

**Keywords:** anthocyanin, smooth muscle cells, metabolism, antioxidant, atherosclerosis

## Abstract

Anthocyanins are reported to have cardio-protective effects, although their mechanisms of action remain elusive. We aimed to explore the effects of microbial metabolites common to anthocyanins and other flavonoids on vascular smooth muscle heme oxygenase-1 (HO-1) expression. Thirteen phenolic metabolites identified by previous anthocyanin human feeding studies, as well as 28 unique mixtures of metabolites and their known precursor structures were explored for their activity on HO-1 protein expression in rat aortic smooth muscle cells (RASMCs). No phenolic metabolites were active when treated in isolation; however, five mixtures of phenolic metabolites significantly increased HO-1 protein expression (127.4–116.6%, *p* ≤ 0.03). The present study demonstrates that phenolic metabolites of anthocyanins differentially affect HO-1 activity, often having additive, synergistic or nullifying effects.

## 1. Introduction

It is now accepted that microbial metabolites (phenolic and aromatic ring-fission catabolites) of dietary flavonoids are more bioavailable than their precursor structures [1]. We have previously reported that anthocyanin metabolites have additive activity in inflammatory systems [2,3], and others have also demonstrated this in vascular smooth muscle cells [4]. As the mechanisms of action of flavonoids have remained unresolved for decades, the need to elucidate the activity of phenolic metabolites has become the focus of recent flavonoid research.

Human feeding studies have shown beneficial effects of flavonoid-rich foods on vascular function, including blood flow and flow-mediated vasodilation (FMD) [5]. Vascular smooth muscle cells contain various sources of reactive oxygen species (ROS), such as NAPDH oxidase (NOX), which under conditions of stress lead to proliferation, migration, and cytokine production, events which are central to the progression of atherosclerosis [6]. We have previously demonstrated that a number of anthocyanin metabolites decreased superoxide ions in cultured endothelial cells but had no effect on the enzyme responsible for their generation, NOX [7]. However, anthocyanin metabolites increased the expression of oxidant-response protein, heme oxygenase-1 (HO-1), which may induce antioxidant activity through the production of biliverdin (converted into cellular antioxidant bilirubin), a by-product of heme degradation [8].

**Figure 1:**
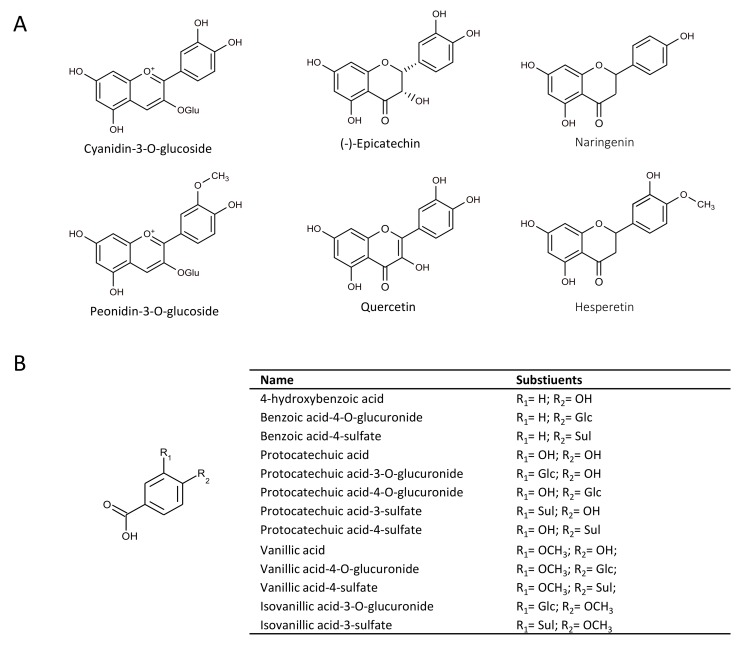
Structures of (**A**) flavonoids and (**B**) metabolites included in treatments. OH, hydroxyl; Glc, oxygen-linked-glucuronide; OGlu, oxygen-linked glycoside; Sul, sulfate; OCH_3_, oxygen-linked methyl group.

In the present study, we therefore explored the action of microbial-derived metabolites of anthocyanins on HO-1 production as a means to rationalise our previous observations. Many of these metabolites are also common to other precursor flavonoids [1]. The aim of the present study was to determine the activity of 13 common phenolic metabolites, identified from human feeding studies [1], relative to six precursor flavonoids (Figure 1), as well as 28 unique mixtures (Table S1), on HO-1 expression in rat aortic smooth muscle cells (RASMCs). 

## 2. Results

### 2.1. Effect of Flavonoids and Their Metabolites on HO-1 Protein Expression

**Figure 2:**
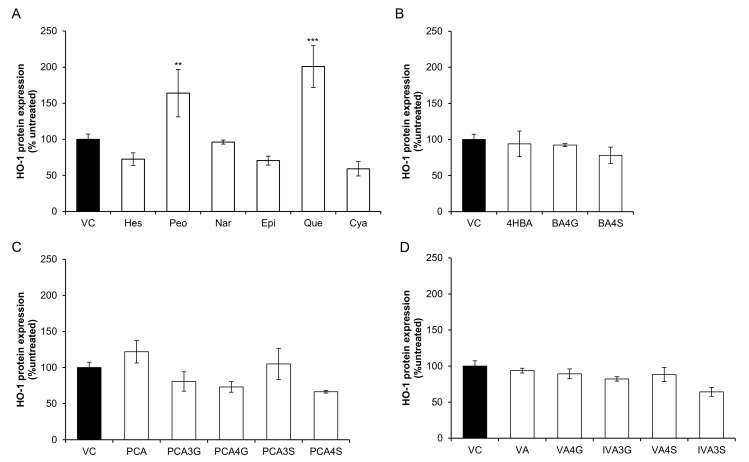
Effect of 10 µM flavonoids and phenolic acid metabolites on HO-1 protein expression in RASMCs. (**A**) precursor flavonoids; (**B**) benzoic acid metabolites; (**C**) protocatechuic acid metabolites; and (**D**) vanillic acid metabolites. Data are presented as a percentage of an untreated control (media only). Filled columns represent vehicle control (0.02% DMSO) and clear columns represent treatments (10 µM). All columns are representative of the mean ± SD, *n* = 3 independent samples, ** *p* ≤ 0.01, *** *p* ≤ 0.001 (ANOVA with post hoc Dunnett relative to vehicle control (0.02% DMSO)). 4HBA, 4-hydroxybenzoic acid; BA4G, benzoic acid-4-*O*-glucuronide; BA4S, benzoic acid-4-sulfate; C3G, cyanidin-3-*O*-glucoside; EPI, (−)-epicatechin; HES, hesperetin; IVA, isovanillic acid; IVA3G, isovanillic acid-3-*O*-glucuronide; IVA3S, isovanillic acid-3-sulfate; NAR, naringenin; P3G, peonidin-3-*O*-glucoside; PCA, protocatechuic acid; PCA3G, protocatechuic acid-3-*O*-glucuronide; PCA4G, protocatechuic acid-4-*O*-glucuronide; PCA3S, protocatechuic acid-3-sulfate; PCA4S, protocatechuic acid-4-sulfate; QUE, quercetin; VA, vanillic acid; VA4G, vanillic acid-4-*O*-glucuronide; VA4S, vanillic acid-4-sulfate.

Thirteen phenolic metabolites and six precursor flavonoids were screened for their effect at 10 µM on RASMC HO-1 protein expression after 24 h incubation (Figure 2). HO-1 expression increased in response to two precursor flavonoids, quercetin (200.87% ± 28.82%, *p* = 0.009) and peonidin-3-glucoside (164.05% ± 32.88%, *p* ≤ 0.001) while no significant activity (*p* > 0.05) was observed for single metabolite treatments. Protocatechuic acid (PCA) appeared to have a modest, but non-significant, effect (121.87% ± 15.47%, *p* = 0.18). 

### 2.2. Effect of Mixtures of Flavonoids and Their Metabolites on HO-1 Expression

**Figure 3:**
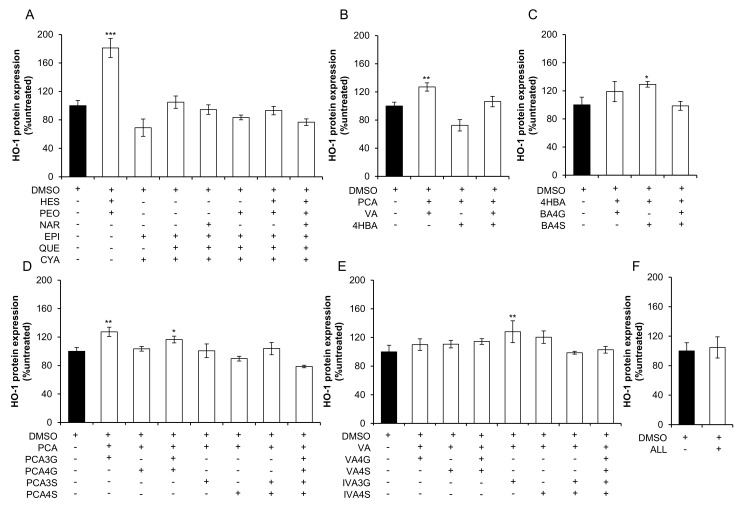
Effect of 10 µM mixtures of flavonoids and phenolic acid metabolites on HO-1 protein expression in RASMCs. (**A**) precursor flavonoids; (**B**) phenolic acids; (**C**) benzoic acid metabolites; (**D**) protocatechuic acid metabolites; (**E**) vanillic acid metabolites; and (**F**) all metabolites. Data are presented as a percentage of an untreated control (media only). Filled columns represent vehicle control (0.02% DMSO) and clear columns represent treatments (10 µM). All columns are representative of the mean ± SD, *n* = 3 independent samples. * *p* ≤ 0.05, ** *p* ≤ 0.01, *** *p* ≤ 0.001 (ANOVA with post hoc Dunnett relative to vehicle control (0.02% DMSO)). ‘ALL’ is a mixture composed of 13 conjugated and unconjugated phenolic acids at equimolar concentrations to a cumulative concentration of 10 µM. 4HBA, 4-hydroxybenzoic acid; BA4G, benzoic acid-4-*O*-glucuronide; BA4S, benzoic acid-4-sulfate; C3G, cyanidin-3-*O*-glucoside; DMSO, dimethyl sulfoxide; EPI, (−)-epicatechin; HES, hesperetin; IVA, isovanillic acid; IVA3G, isovanillic acid-3-*O*-glucuronide; IVA3S, isovanillic acid-3-sulfate; NAR, naringenin; P3G, peonidin-3-*O*-glucoside; PCA, protocatechuic acid; PCA3G, protocatechuic acid-3-*O*-glucuronide; PCA4G, protocatechuic acid-4-*O*-glucuronide; PCA3S, protocatechuic acid-3-sulfate; PCA4S, protocatechuic acid-4-sulfate; QUE, quercetin; VA, vanillic acid; VA4G, vanillic acid-4-*O*-glucuronide; VA4S, vanillic acid-4-sulfate.

Twenty-one mixtures of conjugated and unconjugated phenolic metabolites and seven mixtures of precursor flavonoids, designed based upon their structural similarity, were screened at cumulative concentrations of 10 µM for their effect on RASMC HO-1 protein expression after 24 h treatment (Figure 3). HO-1 expression was increased following treatment with one mixture of precursor flavonoids, consisting of equimolar concentrations of hesperetin and peonidin-3-*O*-glucoside (181.15% ± 13.46%, *p* ≤ 0.001; Figure 3A). Five mixtures of conjugated and unconjugated flavonoid metabolites, including: protocatechuic acid + vanillic acid (127.06% ± 5.83%, *p* = 0.005; Figure 3B); 4-hydroxybenzoic acid + benzoic acid-4-sulfate (129.20% ± 4.00%, *p* = 0.02; Figure 3C); protocatechuic acid + protocatechuic acid-3-*O*-glucuronide (127.43% ± 6.55%, *p* = 0.001; Figure 3D); protocatechuic acid + protocatechuic acid-3-*O*-glucuronide + protocatechuic acid-4-*O*-glucuronide (116.58% ± 4.77%, *p* = 0.03; Figure 3D); vanillic acid + isovanillic acid-3-*O*-glucuronide (128.02% ± 15.26%, *p* = 0.009; Figure 3E), were also active. 

## 3. Discussion

Bacterial catabolism of flavonoids reduces the bioavailability of the precursor flavonoid, while producing a number of bioavailable phenolic metabolites [1]. Notwithstanding previous works suggesting that precursor flavonoids have additive or synergistic effects in combination [2], the present study of the combined effects of flavonoid metabolites is contemporary, owing to the recent availability of synthetic standards. The present study is the first to observe the effects of conjugated phenolic metabolites in combination in vascular smooth muscle cells and suggest that activity of anthocyanins and other flavonoids may be the result of a cumulative effect of multiple metabolites upregulating antioxidant-response protein, HO-1. Here, we found that five mixtures consisting of conjugated and unconjugated phenolic metabolites, one mixture consisting of hesperetin and peonidin-3-*O*-glucoside (Figure 3) and two single flavonoid treatments (quercetin and peonidin-3-*O*-glucoside) (Figure 2) actively upregulated HO-1 protein in RASMCs. These data suggest that conjugated metabolites of flavonoids may not actively increase HO-1 protein in isolation, but act additively or synergistically in combination.

HO-1 protein was increased in response to five mixtures of phenolic metabolites, which is of particular interest as the concentration used (10 µM) is within the range of cumulative metabolite concentrations reported in vivo (0.80–13.18 µM) [9,10]. Keane et al. reported no effect in response to single metabolite treatments protocatechuic acid (PCA) and vanillic acid (VA) on vascular smooth muscle cell (VSMC) migration, whereas mixtures of PCA and VA increased VSMC migration, suggestive of an additive effect [4]. Interestingly, the present study also observed that PCA and VA in isolation did not significantly increase HO-1 expression, but a combination consisting of 5 µM of each metabolite (to a cumulative concentration of 10 µM) significantly upregulated HO-1 protein. This supports the hypothesis that these abundant metabolites act additively on HO-1 expression and provides a rationale for the lack of effect observed in our previous study where no activity was seen in endothelial cells in response to PCA or VA in isolation on protein expression [7]. 

Quercetin significantly induced HO-1 protein expression in the present study, which is in accordance with previous studies, though it should be noted that quercetin circulates as its unconjugated, aglycone structure at negligible concentrations post-consumption [11]. Given that phenolic metabolites exist at much higher concentrations for longer periods of time in the circulation [1], greater focus should be given to their bioactivity in future studies. Similarly, the anthocyanin peonidin-3-*O*-glucoside (P3G) is unstable and rapidly degrades to phenolic acid derivatives at physiological pH and therefore has low plasma bioavailability [10]. The apparent reduction of activity between P3G and its B-ring derivative, VA, suggests that the activity of some anthocyanins may be lost in vivo due to chemical degradation or bacterial catabolism, implying metabolism differentially impacts the activity of anthocyanins. 

The present study has provided a novel insight into the effects of anthocyanin metabolites on HO-1 in RASMCs, though further work is required to elucidate the underlying mechanisms of these treatments. Validation of these effects is required at the mRNA level and including Nrf2 localisation and, ultimately, conformation in animal and human studies. In addition, the use of rat-derived cells may be seen as a limitation, as the phenotypes and expression levels of cellular proteins may not be conserved between species, and, even though costs of using human vascular smooth muscle cells are prohibitive, future work should validate these finding in human cells such as human coronary artery smooth muscle cells (HCASMCs). In addition, the individual 10 µM treatments utilised were beyond the physiological concentrations for single precursor flavonoids [10], but necessary as a comparison to the combination treatments, which were well within physiologically achievable concentrations. In a previously published study, we observed effects on HO-1 expression in response to 10 µM VA in human endothelial cells [7]. Therefore, prior to the present study, a handful of compounds were tested in RASMC at 1, 10, and 100 µM for their effect on HO-1, and 10 µM was identified as most effective (data not shown) and therefore utilised for the present screen. It is important to note that treatments may be more active at concentrations below 10 µM, as in a previous study by our group treatment concentrations as low as 0.19 µM were active [3], and therefore HO-1 expression in response to varied concentrations should be explored in future studies.

## 4. Materials and Methods 

### 4.1. Treatments

The conjugated metabolites: benzoic acid-4-*O*-glucuronide, benzoic acid-4-sulfate, isovanillic acid-3-*O*-glucuronide (4-methoxybenzoic acid-3-*O*-glucuronide), isovanillic acid-3-sulfate (4-methoxybenzoic acid-3-sulfate), protocatechuic acid-3-*O*-glucuronide (4-hydroxybenzoic acid-3-*O*-glucuronide), protocatechuic acid-4-*O*-glucuronide (3-hydroxybenzoic acid-4-*O*-glucuronide), protocatechuic acid-3-sulfate (4-hydroxybenzoic acid-3-sulfate), protocatechuic acid-4-sulfate (3-hydroxybenzoic acid-4-sulfate), vanillic acid-4-*O*-glucuronide (3-methoxybenzoic acid-4-*O*-glucuronide), and vanillic acid-4-sulfate (3-methoxybenzoic acid-4-sulfate), were previously synthesised at the University of St. Andrews (UK) [12]. Flavonoids (cyanidin-3-*O*-glucoside, (−)-epicatechin, hesperetin, naringenin, and quercetin) and unconjugated phenolic acids: 4-hydroxybenzoic acid, protocatechuic acid (3,4-dihydroxybenzoic acid), and vanillic acid (4-hydroxy-3-methoxybenzoic acid), were obtained from Sigma Aldrich (Dorset, UK), with the exception of peonidin-3-*O*-glucoside (Extrasynthase, Genay, France). Stock solutions of all compounds were prepared in 100% DMSO at 200 mM, with the exception of cyanidin-3-*O*-glucoside and peonidin-3-*O*-glucoside, which were prepared at 40 mM, and sulfate-conjugated phenolic acids at 25 mM in 50% DMSO (50% PBS) to maintain stability whilst reducing final DMSO concentrations in working solutions. All stock solutions were stored at 80 °C. Working solutions of individual treatments were added to supplemented media at 10 µM concentrations immediately prior to treatment. Treatments containing mixtures of compounds consisted of equimolar concentrations of the constituent treatment compounds (Table S1) to a cumulative concentration of 10 µM. For example, a combination comprising four constituents required 2.5 µM of each, equating to a total concentration of 10 µM. No treatments were cytotoxic as established utilising the WST-1 cytotoxicity assay (Roche, West Sussex, UK) (data not shown).

### 4.2. Cell Culture

Cryopreserved, second passage, pooled Clonetics rat aortic smooth muscle cells (RASMCs) from adult Sprague Dawley (Lonza Biologics, Manchester, UK), were maintained in Dulbecco’s modified Eagle’s medium: F12 (DMEM) containing 0.1% gentamycin/amphotericin and 20% FBS (Lonza Biologics, Manchester, UK). Cells were used between passages 3 and 6.

### 4.3. HO-1 Protein Expression

RASMC were seeded at 300,000 cells/well in fibronectin-coated 6-well plates. Supplemented media was replaced by serum-free media 24 h prior to experiment commencement. Cells were treated with media only (untreated control), 10 µM treatment, 0.02% DMSO (vehicle control), and incubated for 24 h at 37 °C, 5% CO_2_, in a humidified atmosphere. Cells were washed 3x with PBS and cells lysed with Extraction Reagent Buffer (Enzo Lifesciences, City, UK) and stored at −80 °C until required, undergoing one freeze-thaw cycle. HO-1 protein in cell lysates was determined by use of Rat Hmox-1 ELISA Kits (Enzo Lifesciences, Exeter, UK) according to the manufacturer’s instructions. HO-1 protein concentrations were normalised to the total cell protein content using the Pierce Protein BCA protein assay (Thermo Fisher Scientific, Loughborough, UK).

### 4.4. Data Analysis

HO-1 protein values were presented as a percentage of an untreated control (media only) and reported as the mean ± standard deviation of 3 independent samples. Treatment effects were determined relative to the vehicle control (0.02% DMSO) and established by one-way analysis of variance (ANOVA) with post hoc Dunnett. Analyses were conducted using SPSS for Windows (version 22.0; IBM, New York, NY, USA). Data were considered significant where *p* ≤ 0.05.

## 5. Conclusions

In conclusion, the present study has demonstrated that the bioactivity of common phenolic metabolites is increased when in combination, indicating additive or synergistic effects. 

## Supplementary Materials

The following are available online at http://www.mdpi.com/1420-3049/23/4/898/s1: Table S1: Combination treatment constituents and relative concentrations.
